# Direct measurement and analytical description of the mode alignment in inversely tapered silicon nano-resonators

**DOI:** 10.1038/s41598-019-45034-0

**Published:** 2019-06-21

**Authors:** Sebastian W. Schmitt, Klaus Schwarzburg, Catherine Dubourdieu

**Affiliations:** 1 Institute Functional Oxides for Energy-Efficient Information Technology, Helmholtz - Zentrum Berlin für Materialien und Energie, Hahn-Meitner Platz 1, 14109 Berlin, Germany; 2 Institute for Solar Fuels, Helmholtz - Zentrum Berlin für Materialien und Energie, Hahn-Meitner Platz 1, 14109 Berlin, Germany; 3 Freie Universität Berlin, Physical Chemistry, Arnimallee 22, 14195 Berlin, Germany

**Keywords:** Nanocavities, Silicon photonics

## Abstract

Inversely tapered silicon photonic resonators on silicon substrates were shown to host multiple high–Q whispering gallery modes and constitute versatile building blocks for CMOS compatible solid state lighting, optical sensing and modulator devices. So far, numerical analyses by the finite difference time domain method have been used to predict the height distribution of whispering gallery modes in such resonators. In this study, we provide an experimental evidence of this mode distribution along the resonator height by selectively exciting whispering gallery modes using cathodoluminescence spectroscopy. Further we derive analytical functions that permit to relate the height distribution of modes with a defined polarization, symmetry and effective refractive index to the geometrical shape of the inversely tapered resonators.

## Introduction

The emerging research field of silicon (Si) nanophotonics is pointing out promising ways towards revolutionary new devices that rely on the nonlinear interaction of light with Si nanostructures^1–4^. Modern Si nanophotonic waveguides and optical modulators pave the way towards on-chip optical data transfer that is faster and more energy efficient than existing electrical technologies^5,6^. By confining light into modes, Si nanostructures can enhance light absorption^7,8^, which led to the fabrication of optimized nano-patterned Si-based optical absorbers for detectors, sensors and solar cells^9–11^ while the evanescent field of confined photonic modes could be used for highly sensitive small scale medical, biological or chemical sensing devices^12–17^. Furthermore it could be shown that the confinement of spectrally matched modes amplifies spontaneous emission rates of optical transitions in Si nanophotonic cavities, which could successfully be applied to improve the performance of Si-based light emitting devices (LEDs)^18,19^. Hereby, the amplification is proportional to Q/V, where Q is the quality factor of the cavity and V is the volume of the resonant mode (Purcell effect)^20^.

Inversely tapered Si photonic resonators were shown to enhance photoluminescence and light absorption in a multitude of photonic whispering gallery modes (WGMs)^21,22^. Using finite difference time domain (FDTD) simulations, the height distribution of the modes could be analyzed, but so far, its direct experimental proof as well as its relation to the geometrical shape of the resonators is missing. Cathodoluminescence (CL) spectroscopy permits to experimentally visualize luminescent modes in photonic resonators and has already been used in e.g. the analysis of the mode distribution in photonic crystal cavities^23–27^. In this study, we selectively excite the modes of inversely tapered Si photonic resonators using CL spectroscopy, to experimentally map the WGMs in such integrated photonic resonators on Si. Further, we derive analytical functions, which directly relate the height distribution of modes with a specific symmetry, effective refractive index and polarization to the geometrical shape of the inverse taper of the resonator. The study is performed on a Si resonator with an inverse ellipsoidal taper that has not been shown so far (inverted half silicon nano- ellipsoid: SiNE). In the Supplementary Information, the analysis is repeated for a Si resonator with an inverse conical taper (inverted silicon nanocone: SiNC) to show the generality of the described concept.

## Results and Discussion

**Figure 1:**
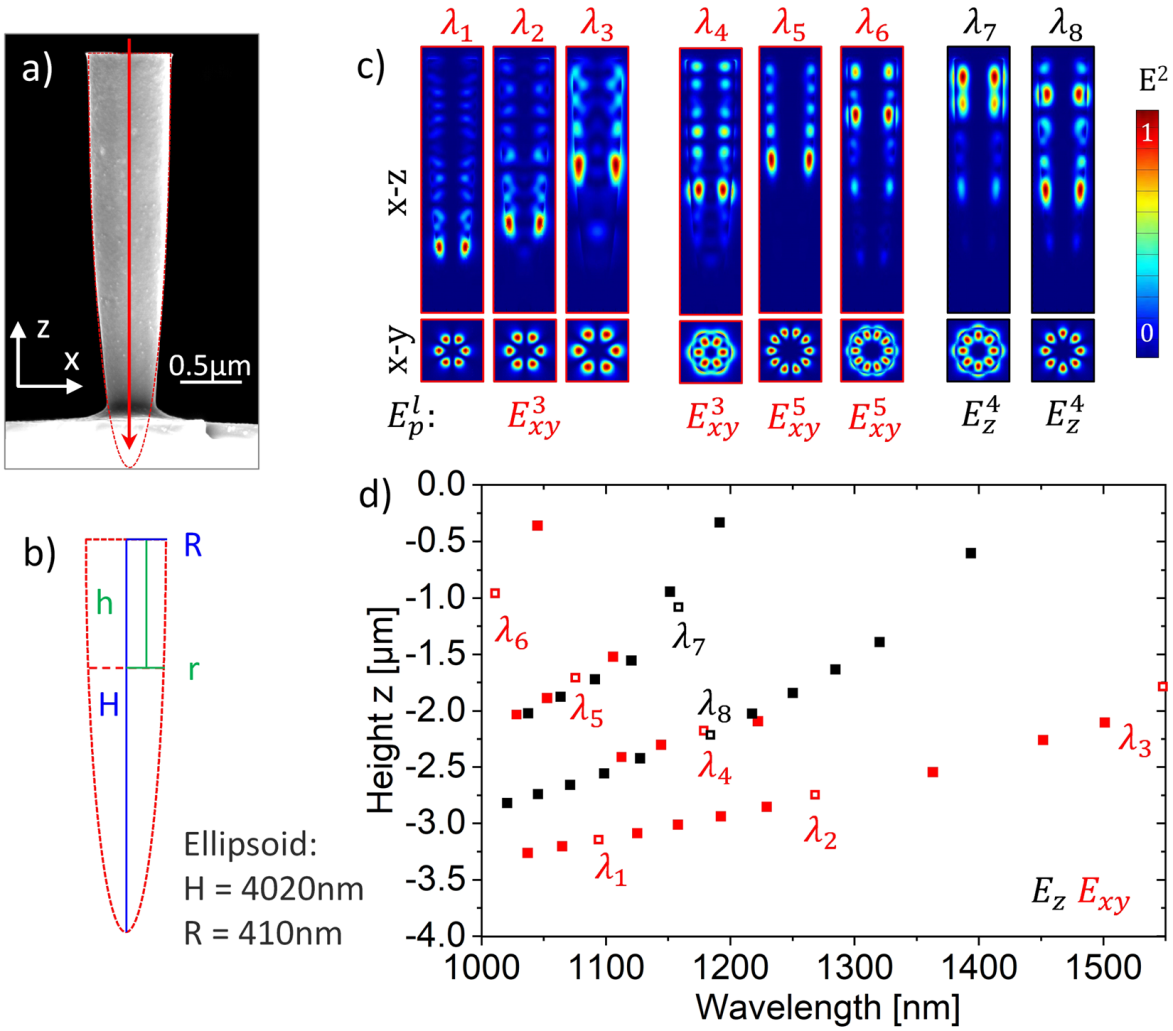
(**a**) SEM image of a SiNE photonic resonator. The red dotted line indicates the ellipsoidal outer shape of the resonator geometry. The red arrow indicates the line scan of the electron beam in the CL measurement (along – z direction/compare Fig. 2). (**b**) Cross-sectional geometry with characteristic dimensions of the SiNE as shown in (**a**). **(c)** FDTD simulations of the relative cross sectional energy density (E^2^) in x-y and x-z direction for all six different types of photonic modes found in the SiNE. The nomenclature ${E}_{p}^{l}$ describes the properties of six mode types. Here, *p* is the polarization of the electric field (in x-y direction or along z direction) and *l* the number of the radial symmetry. **(d)** Occurrence height of photonic modes (WGMs) in a SiNE versus wavelength determined by numerical FDTD simulations as given in Fig. 1c. Red squares correspond to modes polarized in x-y direction while black squares correspond to modes polarized in z-direction. Open squares indicate modes *λ*_1_–*λ*_8_ for which the relative cross-sectional energy density (*E*^2^) in x-y and x-z direction is given in panel 1c. The z = 0 position is at the top of the resonator.

A scanning electron microscopy (SEM) image of an as-fabricated SiNE resonator is presented in Fig. 1a. Figure 1b shows the cross-sectional geometry of a SiNE with the dimensions for (total) height H and upper radius R taken from Fig. 1a. The parameters r and h indicate radius and height at an arbitrary position inside the resonator. Figure 1c shows FDTD simulations of the relative cross sectional energy density (*E*^2^) in x-y and x-z direction for eight exemplary photonic modes at wavelengths *λ*_1_ = 1088 nm, *λ*_2_ = 1264 nm, *λ*_3_ = 1541 nm, *λ*_4_ = 1172 nm, *λ*_5_ = 1069 nm, *λ*_6_ = 1004 nm, *λ*_7_ = 1145 nm *λ*_8_ = 1171 nm in a SiNE with dimensions as indicated in Fig. 1b. It can be seen that modes in the SiNE resonator can be identified with WGMs that are confined to discrete orbits by a ‘leaky’ Fabry-Perot branch towards the top surface. Further, the numerical simulations reveal that the modes have a polarization *p* in x-y plane (indicated in red) or along the z-axis of the resonators (indicated in black) and exhibit a 3, 4 or 5 – fold symmetry *l* in the x-y plane as expressed by the label ${E}_{p}^{l}$. Each of the three symmetries is degenerate in two types of WGMs with a different intensity of the evanescent field surrounding the structure (see Fig. 1c modes *λ*_1_–*λ*_3_ and *λ*_4_, modes *λ*_5_ and *λ*_6_, modes *λ*_7_ and *λ*_8_). Accordingly, six (3 symmetries degenerate in 2 types of evanescent field) different types of modes were found. For different wavelengths modes of a specific type can appear in different heights of the SiNE resonator (compare Fig. 1c modes *λ*_1_, *λ*_2_, *λ*_3_). The cross-sectional energy density (*E*^2^) of all photonic modes in the SiNE with a quality factor Q > 200 was determined by FDTD simulations and the orbit height of the corresponding WGMs was plotted against the mode wavelength in Fig. 1d. As in Fig. 1c modes polarized in x-y plane are indicated in red color while modes polarized along the z-axis are indicated in black. It is found that sets of modes align along curved branches, each of which can be identified with one of the six mode types in Fig. 1c.

**Figure 2:**
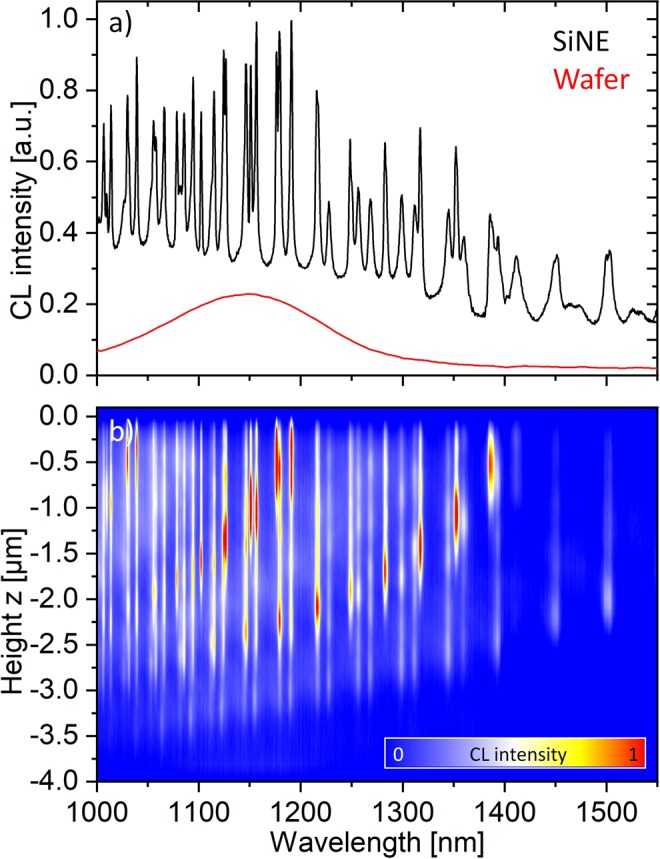
(**a**) Average CL emission of a SiNE resonator measured along the line scan shown in Fig. 1a. The red line shows the emission of a planar Si wafer for reference. (**b**) Intensity map of the height selective CL emission of the SiNE) along the line scan shown in Fig. 1a. The z = 0 position is at the top of the resonator.

Figure 2a shows the average CL emission of the SiNE along the scan path shown in Fig. 1a. A multitude of sharp emission peaks is observed. The red line shows the emission of a planar Si wafer collected with the same integration time. Compared to room temperature photoluminescence (PL) from the SiNEs (see Supplementary Information S2) and Si photonic resonators from previous studies, we observe that the CL emission extends far beyond 1250 nm^21,28,29^. While the enhanced emission up to 1250 nm can be explained by a broadened Si band edge emission at high temperatures, the emission above this value has a different origin. For the Si wafer with a resistivity of 1–5Ωcm (red line in Fig. 2a) the shallow radiation for wavelength > 1250 nm can be attributed to coherent CL emission. The SiNE has a similar doping and it is measured at the same excitation energy, however the emission in the range between 1250 and 1550 nm is much higher than for the Si wafer. As compared to the planar single side polished Si wafer, the Si resonators fabricated by reactive ion etching have considerably more surface defects and, due to their geometry, an inferior thermal coupling to the substrate. Accordingly, a much stronger incoherent emission from radiative surface defect states or a higher thermal radiation due to a lower heat dissipation can account for the significantly higher CL signal in the range between 1250 and 1550 nm^23,30–33^. This argument is further supported by additional CL spectra recorded on an individual SiNE for different acceleration voltages (5, 15 and 30 keV). While in case of coherent emission the CL signal for the higher energy electrons should be significantly enhanced, our experiments show a comparable intensity of the CL signal for high and low acceleration voltages (Supplementary Information S4). This can be attributed to a comparable interaction volume of the electron beam with the SiNE for high and low acceleration voltages, which would generate a comparable incoherent emission intensity^34^.

Figure 2b gives the CL intensity distribution in the SiNE modes when using a height selective excitation by the electron beam (scan step 100 nm/scanning direction is z-direction as shown in Fig. 1c). We observe that the strongest emission for any of the peaks shown in Fig. 2a is closely linked to a distinct height in the SiNE resonator, which is in line with the presence of WGMs in the inversely tapered resonators as determined by FDTD. Further, the emission of all detected modes faintly extends towards the top and bottom surfaces of the resonators, which coincides with the leaky branches in the numerical simulations from Fig. 1c. In the WGMs, the resolution of the CL measurements appears to be lower than ~250 nm, which is the resolution estimated by Monte Carlo simulations of the electron trajectories in the experimental setup (see methods and Supplementary Information S3). This can be attributed to a low diffusion length in the Si resonators due to a high surface recombination velocity, and a strongly localized amplification of radiation inside the detected photonic modes.

**Figure 3:**
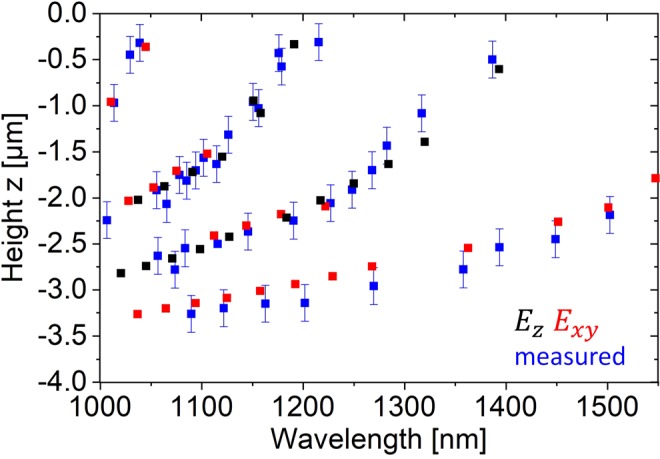
Superposition of the numerically simulated (red E_xy_/black E_z_ rectangles) and measured (blue rectangles) occurrence height of photonic modes (whispering gallery modes) in a SiNE versus wavelength. The z = 0 position is at the top of the resonator.

Heights of the WGMs in the resonator were extracted from the CL intensity map (Fig. 2b) by determining the height of the CL intensity maximum for each wavelength. In Fig. 3, the height distribution of the modes determined from the CL measurements is compared to the simulated height distribution of the WGMs from the FDTD simulations. An excellent coincidence is observed between measured and numerically simulated data considering the experimental error (~250 nm) that was estimated by the Monte Carlo simulations and that physically resembles the convolution of the height selective CL signal with the size of the excitation plume from the electron beam. Note that the samples were dipped into HF prior to CL measurements in the SEM, in order to remove native SiOx oxide that would influence the photonic modes hosted by the resonators. In contrast to mode positions and wavelength, average experimentally determined Q-factors (200–800) ranged about one order of magnitude below the ones determined from the FDTD simulations. This can be explained by mode damping due to scattering on surface imperfections of the experimentally measured resonators. In experiment and simulations, we find a tendency towards higher Q-factors in higher orbits of the structures. This can be attributed to a lower edge scattering and bending loss for WGMs in orbits of a higher radius which has been described for WGM micro disk resonators^33^.

In Fig. 3 it can be seen that the experimentally measured and numerically determined modes - plotted over the wavelength - align along branches in height, each of which can be associated with one of the six mode types described in Fig. 1c. In the following it will be shown that shape and position of these branches follow analytical functions which can relate the geometry of inverse taper of the resonator to the height alignment of associated WGMs with specific properties. Following Fig. 1b, an arbitrary radius *r* ≤ *R* in the SiNE is related to a corresponding height *h*, by the following relation:

1 $$h(r)=\frac{H}{R}\sqrt{{R}^{2}-{r}^{2}}$$

WGMs in an orbit with radius *r*, can be described by the well-known relation

2 $$r=\frac{l\cdot \lambda }{2\pi {n}_{e}}$$

with *n*_*e*_ being the effective refractive index and *l* the radial symmetry (here 3, 4, or 5) and λ the wavelength of the resonant modes^35^.

Combining (1) and (2) results in

3 $$h(\lambda )=\frac{H}{R}\sqrt{{R}^{2}-{(\frac{l\cdot \lambda }{2\pi {n}_{e}})}^{2}}$$

for the height of the WGM orbit in the SiNE.

**Figure 4:**
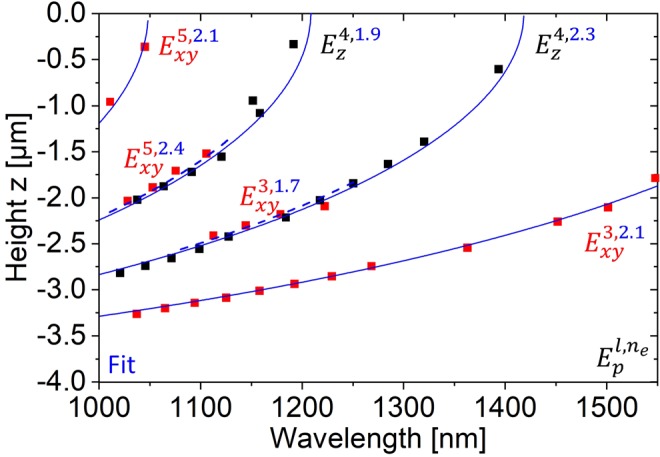
Occurrence height of photonic modes (WGMs) in a SiNE versus wavelength as in Fig. 1d. Blue lines (dashed and solid for visual discrimination of adjacent lines) correspond to fits of Eq. 3 to the simulated data that provide the effective refractive index *n*_*e*_of the mode branches. The nomenclature ${E}_{p}^{l,{n}_{e}}$ corresponds to the one used in Fig. 1c, complemented by *n*_*e*_ from the fit of Eq. 3. The z = 0 position is at the top of the resonator.

Equation (3) was used to fit the data in Fig. 1d with the effective refractive index *n*_*e*_ as the only free parameter and Fig. 4 shows that excellent fits are obtained using the analytical formula. Note that while the analytical fits suggest a continuous distribution of WGMs along the resonator height, the actual presence of WGMs in discrete orbits is related to the simultaneous occurrence of a Fabry-Perot mode between the WGMs and the top facet of the resonator.

A mode branch is fully characterized by the label ${E}_{p}^{l,{n}_{e}}$, which is the label in Fig. 1c for WGMs with different wavelength λ and specific polarization *p* and symmetry *l* complemented by the effective refractive index *n*_*e*_ derived from the analytical approach. Effective refractive indices n_e_ for the six mode branches fitted in Fig. 4 were found to be 1.7, 1.9, 2.1, 2.1, 2.3 and 2.4. By definition of our analytical model, these values are constant over the entire wavelength range of the fit and their magnitude is in line with the observation of modes overlapping the boundary between Si (n = 3.5) and vacuum (n = 1). Modes with *n*_*e*_ > 2 compare well to literature values of Si disk resonators. However, the Q-factors of disk resonators are several orders of magnitude higher^36^. From the cross-sectional energy density plots in Fig. 1c, it can be seen that mode branches with an effective refractive index *n*_*e*_ < 2 correspond to WGMs that have a more intense evanescent field i.e. have a higher overlap with the medium surrounding the resonator (here: vacuum with *n* = 1). In particular, this becomes evident when comparing e.g. ${E}_{z}^{4,1.9}\,$and ${E}_{z}^{4,2.3}$ that show the same (4-fold) symmetry but a different refractive index. Further, comparing the branches ${E}_{xy}^{5,2.4}$, ${E}_{z}^{4,1.9}$ and ${E}_{z}^{4,2.3}$, ${E}_{xy}^{3,1.7}$, it can be observed that branches (and even some modes on those branches) with different polarization, refractive index and symmetry almost coincide in the graphs (and accordingly some of the associated modes occupy overlapping positions in the resonators).

The CL investigation and analytical analysis of the photonic modes was repeated for Si nanocones (SiNC), which were previously analyzed by PL and numerical FDTD modeling^21^. The results are presented in Figures S1–S4 in the Supplementary Information S1. The CL measurements as a function of height along nanocones confirm again the presence of WGMs at given heights that match the one determined by FDTD modeling. The analytical formula derived for the nanocones - where only the characteristic dimensions of the SiNC resonator and the refractive index of Si are introduced – describe very well the branches of the different mode types. One can note that the modes in nanocones (SiNC) align along linear branches while Si half-nano ellipsoids (SiNE) have curved branches, which is a direct consequence of the geometry of the resonators as clearly shown by the analytical approach. Accordingly, the analytical approach proposed in this paper can be extended to any inversely tapered photonic resonator geometry.

## Conclusions

In the presented study, we experimentally determine the height alignment of WGMs in inversely tapered Si photonic resonators by their selective excitation using CL spectroscopy. We show an excellent agreement with the height alignment derived from FDTD simulations. Further we show that the height alignment of WGMs with a similar symmetry, polarization and effective refractive index in inversely tapered Si photonic resonators follows analytical functions that can be derived from the geometrical shape of the inverse taper of the resonator. The experimental confirmation of WGMs as well as the theoretical understanding of the inversely tapered Si resonators combining an analytical approach to the FDTD one provide a novel platform for the design of Si on-chip photonic devices.

## Methods

The SiNE photonic resonators were fabricated using cryogenic reactive ion etching (RIE/SF_6_ – O_2_ plasma) of a Si wafer masked by polystyrene nanosphere lithography. The Si wafer used for fabrication was single-side polished (100) and boron-doped with a nominal resistivity in the range of 1–5 Ωcm. Starting from a recipe for straight sidewalls, recipes with lowered process temperatures and O_2_ fluxes were used, to achieve the different forms of inverse surface taper^21^. After the etching, the resonators underwent a surface post treatment consisting of an oxidation step of 10 min at 500 °C in an O_2_ atmosphere and an HF dip in 1% aqueous solution. The HF dip was performed directly before the introduction of the samples into the SEM chamber, to avoid the formation of a SiO_2_ surface layer.

For the mode analysis (Lumerical FDTD), broadband dipole pulses (950–1600 nm) polarized in x-y and z direction (E_xy_, E_z_) were excited in a SiNE and a SiNC with the geometries described in Fig. 1c,d and supplementary Fig. S1c,d. After an apodization time of 1100 fs the remaining sideward emission from the SiNE an the SiNC was determined, and converted to a spectral energy distribution via a Fourier transformation (for details see also ref.^21^). For each peak in the spectrum, the height of the corresponding WGM was derived from the x-z cross sectional energy density (E^2^) map as plotted in Figs 1 and S1. Further, the peaks were fitted with a Lorentzian profile and the spectral position (λ) the full width half maximum (FWHM) and the Q – factor of the simulated modes was determined (Q = λ/FWHM).

CL measurements were performed in an SEM (ZEISS Merlin) equipped with CL system (DELMIC Sparc). The system was equipped with an Andor Kymera 193i spectrometer (300 l/mm grating blazed at 1200 nm) and an Andor iDus InGaAs array (−60 °C), which accounts for an absolute intensity error of about ± 10% in the measured spectral range between 1000 and 1550 nm. Since we were interested only in the positions and widths of the spectral peaks, we did not apply an intensity correction. Line scans along the SiNE under a 90° tilt view were executed, using a beam energy of 7 keV, a beam current of 4.4 nA, a step size of 100 nm and an integration time of 12 s. While the electron beam scans the height of the resonator and excites the different modes, the focus of the parabolic mirror stays in the same position. Furthermore, the far-field emission of the resonators depends on the type (polarization, symmetry, effective refractive index) of the excited mode. This means that, under the present experimental conditions, the CL collection efficiency of a mode (i.e. its relative spectral intensity) can slightly be affected by the type of the excited mode relative to efficiency of the analytical optical path and the position of the mode in the resonator relative to the focus of the parabolic mirror. The peaks in the acquired spectra were fitted with a Lorentzian profile and the spectral position (λ) the full width half maximum (FWHM) and the Q – factor of the measured modes was determined (Q = λ/FWHM). 3D Monte Carlo simulation of an electron beam penetrating a Si nanowire of 500 nm provided the interaction volume of the electron beam with the Si resonators, showing that for the instrumental settings applied in this study a lateral resolution of the CL excitation of <250 nm can be estimated. The CL line scans were collected using a step size of 100 nm and an integration time of 12 s (see Supplementary Information S3 for a sketch of the setup and the results of the Monte Carlo simulation).

## Supplementary information

Supplementary Information

## Data Availability

All experimental data and parameters used for fittings and FDTD modeling are available upon request.
