# Supplementary material for: Direct measurement and analytical description of the mode alignment in inversely tapered silicon nano-resonators
Source: Sci Rep. 2019 Jun 21;9:9024. doi: 10.1038/s41598-019-45034-0 (PMC6588582; doi:10.1038/s41598-019-45034-0)
Supplement: Supplementary file 1 — Supplementary Information [file 41598_2019_45034_MOESM1_ESM.pdf]

Supplementary information: Direct measurement and analytical description of the mode alignment in inversely tapered silicon nano-resonators

Sebastian W. Schmitt,[1*] Klaus Schwarzburg[2] and Catherine Dubourdieu[1,3*]

[1] Institute Functional Oxides for Energy-Efficient Information Technology, Helmholtz - Zentrum Berlin für Materialien und Energie, Hahn-Meitner Platz 1, 14109 Berlin, Germany

[2] Institute for Solar Fuels, Helmholtz - Zentrum Berlin für Materialien und Energie, Hahn-Meitner Platz 1, 14109 Berlin, Germany

[3] Freie Universität Berlin, Physical Chemistry, Arnimallee 22, 14195 Berlin, Germany

* sebastian.schmitt@helmholtz-berlin.de, catherine.dubourdieu@helmholtz-berlin.de

Supplementary information S1:

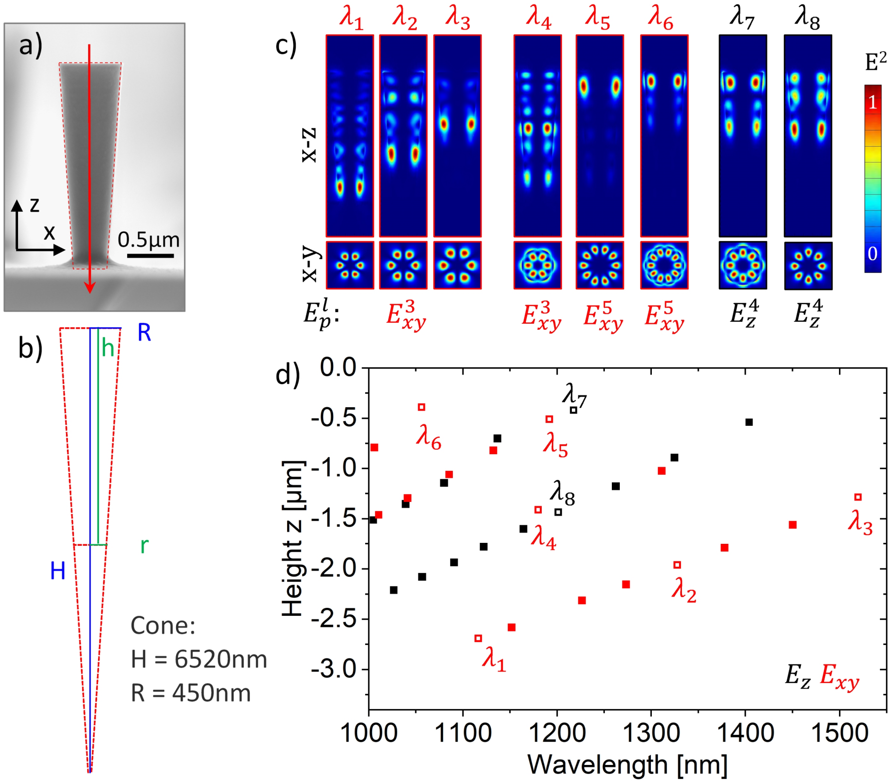

Figure S1. (a) SEM image of a SiNC photonic resonator. The red dotted line indicates the ellipsoidal outer shape of the resonator geometry. The red arrow indicates the line scan of the electron beam in the cathodoluminescence measurement (along – z direction / compare Figure S2). **(b)** Cross-sectional geometry with characteristic dimensions of the SiNC as shown in (a). **(c)** FDTD simulations of the relative cross-sectional energy density (E^2) in x-y and x-z direction for all six different types of photonic modes found in the SiNC. The nomenclature E_p^l

describes the properties of six mode types. Here, *p* is the polarization of the electric field (in x-y direction or along z direction) and *l* the number of the radial symmetry. **(d)** Occurrence height of photonic modes (WGMs) in a SiNC versus wavelength determined by numerical FDTD simulations as given in Figure 1c. Red squares correspond to modes polarized in x-y direction while black squares correspond to modes polarized in z-direction. Open squares indicate modes λ_1 - λ_8 for which the relative cross sectional energy density (E^2) in x-y and x-z direction is given in panel 1c. The z=0 position is at the top of the resonator.

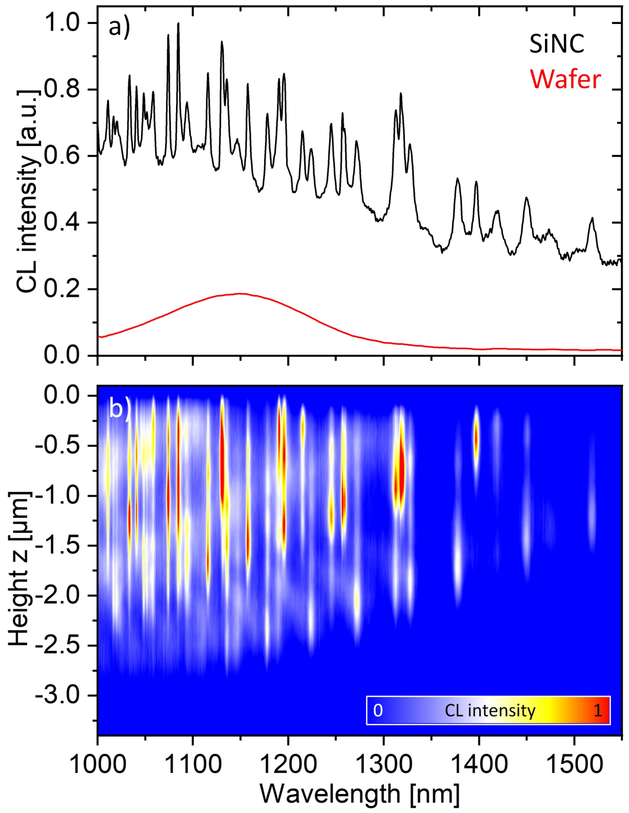

Figure S2. (a) Average CL emission of a SiNC measured along the line scan shown in Figure 1a. The red lines show the emission of a planar Si wafer for reference. **(b)** Intensity map of the height selective CL emission of the SiNC along the line scan shown in Figure S1a. The z=0 position is at the top of the resonator.

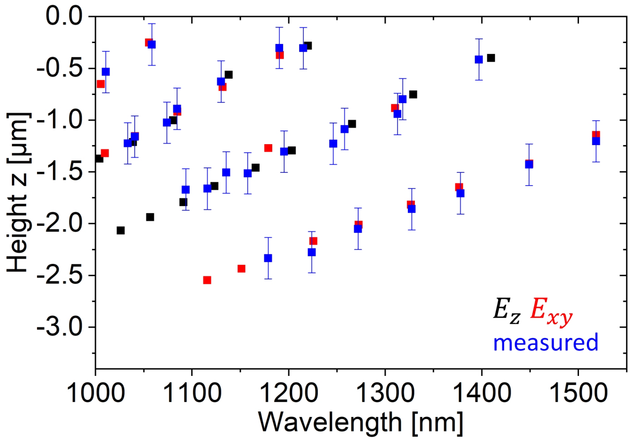

Figure S3. Superposition of the numerically simulated (red E_{xy} / black E_z rectangles) and measured (blue rectangles) occurrence height of photonic modes (whispering gallery modes) in a SiNC versus wavelength. The z=0 position is at the top of the resonator.

Following Figure S1b, an arbitrary radius $r \leq R$ in the SiNC is related to a corresponding height h, by the following relation:

$$h(r) = H - \frac{H}{R} \cdot r \qquad (S1)$$

For the WGMs in an orbit with radius r, we get

$$r = \frac{l \cdot \lambda}{2\pi n_e} \qquad (S2)$$

with n_e being the effective refractive index and l the radial symmetry (here 3, 4, or 5) of the resonant modes. Combining (1) and (2) results in

$$h(\lambda) = H - \frac{H}{R} \cdot \frac{l \cdot \lambda}{2\pi n_e} \qquad (S3)$$

for the height of the mode in the SiNC. Equation (S3) was used to fit the data in Figure S1d with n_e as the only free parameter.

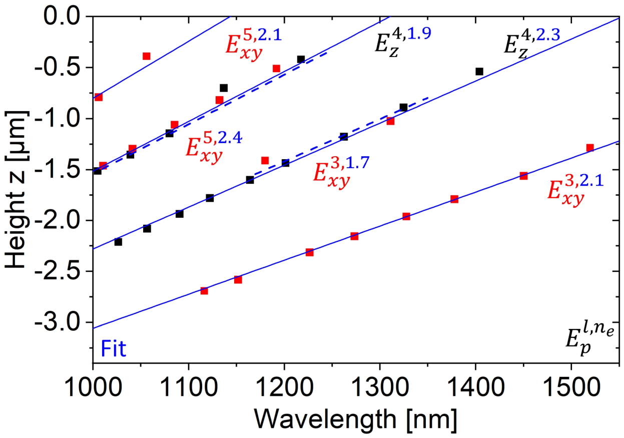

Figure S4. Occurrence height of photonic modes (WGMs) in a SiNC versus wavelength as in Figure 1d. Blue lines (dashed and solid for visual discrimination of adjacent lines) correspond to fits of Equation S3 to the simulated data that provide the effective refractive index n_e of the mode branches. The nomenclature E_p^{l,n_e} corresponds to the one used in Figure 1c, complemented by n_e from the fit of Equation S3. The z=0 position is at the top of the resonator.

Supplementary information S2:

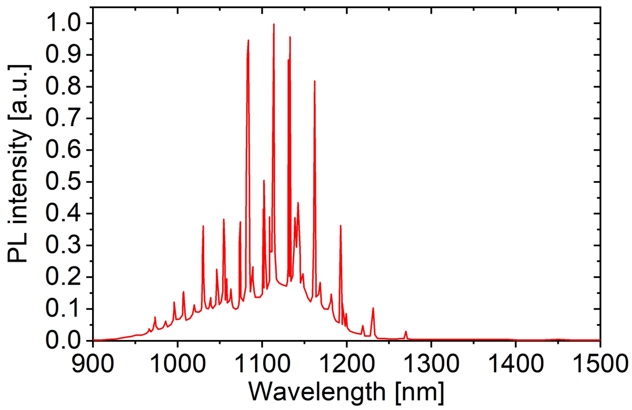

Figure S5. Relative PL intensity from a SiNE at room temperature excited by a 633nm CW laser with a power of 1mW and an integration time of 2x5s.

Figure S5 shows the relative PL intensity of a SiNE at room temperature. Emission is centered around the Si band gap (1100nm) and does not extend far beyond 1250nm.

Supplementary information S3:

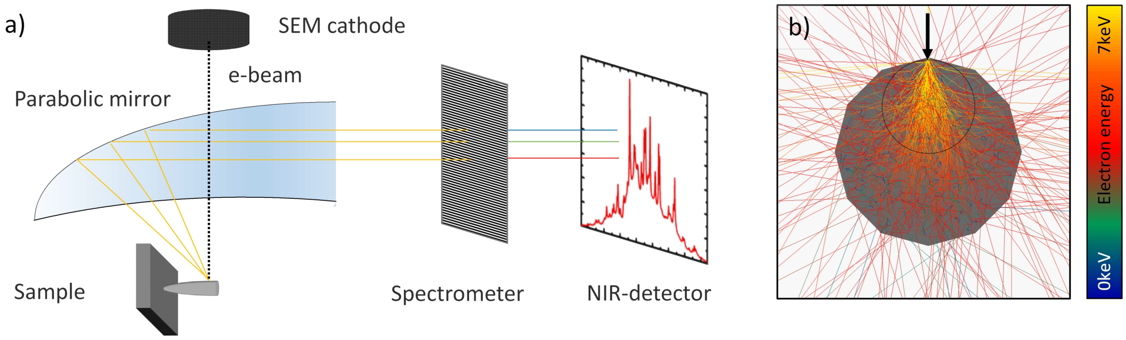

Figure S6. (a) Schematic of the CL setup inside the SEM. **(b)** Cross section of a 3D Monte Carlo simulation of an electron beam (7keV) penetrating a Si nanowire of 500nm diameter. The black line indicates the estimated size of the interaction volume, the black arrow shows the point of incidence of the electron beam.

Measurements where performed with an Andor Kymera 193i spectrometer (300l/mm grating blazed at 1200nm) and an Andor iDus InGaAs array (-60°C). While the efficiency of the grating linearly decreases for about 20%, the response of the InGaAs array is constant in the range between 900 and 1600nm. This accounts for an absolute intensity error of about ± 10% in the measured spectral range between 1000 and 1550nm. No intensity correction was applied to the measurement data, since we were interested only in the spectral positions and widths of the measured peaks. The 300l/mm grating provides a spectral resolution of about 0.5nm, which was sufficient to experimentally determine the width and position of the peaks with the accuracy necessary for the study.

Supplementary information S4:

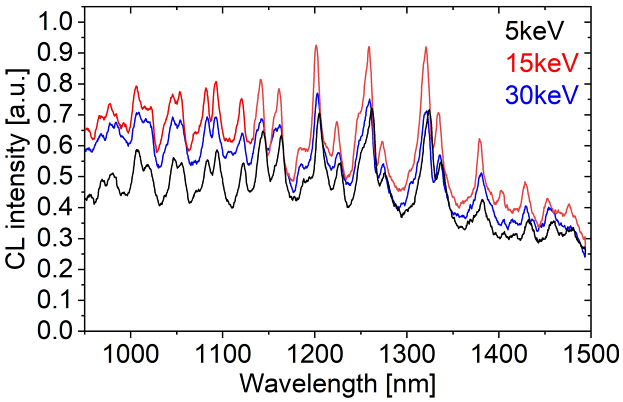

Figure S7. CL spectra recorded on an individual SiNE for different acceleration voltages (5, 15 and 30keV).

Figure S7 shows CL spectra recorded on an individual SiNE for different acceleration voltages (5, 15 and 30keV / beam current 2nA). Spectra are averaged from 10 spectra recorded in a height scan (step size 300nm / integration time 25s) of a SiNE on the same sample as the SiNE shown in Figure 1a. Note that even though both resonators show a different formation of modes, they are fabricated from the same material with the same processes. Therefore mechanisms responsible for the CL emission can be regarded as similar.
